# The tracking and reducing alcohol consumption (TRAC) intervention for veterans living with HIV/AIDS: results from a pilot randomized waitlist-controlled trial

**DOI:** 10.1186/s13722-025-00631-5

**Published:** 2025-12-03

**Authors:** Carolyn Lauckner, Reuben Adatorwovor, Erica Taylor, Fidelis Sesenu, Tehquin Tanner, Alexis Whitmire, Vincent Marconi, Trace Kershaw, Nathan Hansen

**Affiliations:** 1 C.S. Mott Department of Public Health, Michigan State University, 200 East 1st St, Flint, MI 48502 USA; 2 Department of Biostatistics, University of Kentucky, Lexington, KY USA; 3 Interwork Institute, College of Education, San Diego State University, San Diego, CA USA; 4 Center for Health, Engagement, and Transformation, University of Kentucky, Lexington, KY USA; 5 Atlanta Veterans Affairs Medical Center, Decatur, GA USA; 6 Division of Infectious Diseases, Emory University School of Medicine, Atlanta, GA USA; 7 Department of Social and Behavioral Sciences, Yale University, New Haven, CT USA; 8 Department of Health Promotion and Behavior, University of Georgia, Athens, GA USA

**Keywords:** Alcohol misuse, HIV/AIDS, mHealth, Medication adherence, Veterans, Motivational interviewing

## Abstract

**Background:**

Veterans with HIV/AIDS (VWH) frequently report alcohol misuse, which can impact antiretroviral therapy (ART) adherence and lead to poorer clinical outcomes. The TRAC (Tracking and Reducing Alcohol Consumption) intervention was developed to help VWH reduce alcohol use and its associated consequences. TRAC is delivered via mobile device, incorporates eight counseling sessions based in cognitive behavioral therapy and motivational interviewing, and utilizes mobile surveys and breathalyzers for daily monitoring of alcohol and ART use.

**Methods:**

We conducted a pilot randomized waitlist-controlled trial (*N* = 50). Participants were allocated to an immediate intervention group (*N* = 26), which received the TRAC intervention and completed twice-daily monitoring of alcohol and ART use for 8 weeks, or to a waitlist-control (*n* = 24), which started TRAC after 8 weeks. Participants provided ratings of intervention sessions and completed questionnaires assessing alcohol use, ART adherence, and treatment self-efficacy at baseline, 8, 16, and 24 weeks. Analyses included correlations and descriptive statistics for examining feasibility and acceptability, difference-in-differences analyses to compare changes between groups at the 8-week timepoint, matched pair tests to assess changes in alcohol use during the intervention, and general linear models to investigate long-term effects on outcomes with a pooled sample.

**Results:**

Results indicated high feasibility and acceptability: 84% of participants were retained through the intervention and all follow-ups, average intervention session ratings were 9.6 (out of 10), and participants completed a median of 85% and 78% of mobile surveys and breathalyzer readings, respectively. While not statistically significant due to low power, there was a trend of decreased binge drinking episodes and fewer missed HIV medication doses in the intervention group compared to control. When pooling data among participants from both groups to examine long-term effects, TRAC was associated with reductions in several drinking-related outcomes.

**Conclusions:**

High acceptability and feasibility, as well as preliminary evidence that the intervention may reduce alcohol use relative to control, suggest that the TRAC intervention is promising for VWH and warrants further evaluation in a randomized controlled trial with adequate power to detect effects. If shown to be efficacious, TRAC has potential to be a highly scalable and acceptable intervention for delivery among VWH.

**Trial registration:**

This study was registered on ClinicalTrials.gov, #NCT03746600. Registration date: 09/24/2018.

## Background

In the United States, the prevalence of HIV among Veterans is higher than that of the general population [1, 2], and the Department of Veteran’s Affairs (VA) is the largest provider of HIV care in the country. Among Veterans, those diagnosed with HIV are more likely to have alcohol-related conditions in their medical chart and more severe alcohol misuse than those without HIV [3, 4], with approximately 15% of veterans reporting an alcohol use disorder [5]. This has significant implications for HIV care-continuum outcomes, as alcohol misuse among people with HIV is associated with decreased antiretroviral therapy (ART) adherence [6–9], higher risk of virologic failure [9], faster disease progression [6, 10], higher risk of hospitalization [3], and increased HIV transmission risk [11]. A recent study found that HIV viral suppression rates among those with very high levels of alcohol use were just 37%, compared to 58% for nondrinkers and 56% for light drinkers [12]. Unfortunately, veterans with HIV/AIDS (VWH) are 17% less likely to receive alcohol-related care after screening positive for alcohol misuse than those without HIV, highlighting persistent service-delivery gaps [4]. Within the VA system, HIV-positive patients incur substantially greater healthcare costs than matched non-HIV controls; approximately four-fold as many costs in total spending, about 17-fold in pharmacy, and nearly two-fold in outpatient care [13]. Alcohol problems add further utilization burden among veterans, with adjusted incidence rate ratios of 2.17 for outpatient visits, 1.46 for emergency department visits, and 1.46 for inpatient hospitalizations; these rates are significantly higher among VWH with alcohol problems compared to VWH without alcohol problems [14]. For these reasons, intervention approaches that aim to reduce alcohol use and increase ART adherence among VWH are needed.

Several studies of alcohol reduction interventions for people with HIV have yielded mixed results. Multiple reviews of these interventions have been conducted, with three finding limited or mixed evidence for effects on alcohol use [15–17]. However, a 2017 meta-analysis found beneficial effects of behavioral interventions on reducing quantity of alcohol use and heavy drinking episodes, as well as on increasing medication adherence [18]. Importantly, these syntheses note that benefits have been observed across both single-session and multi-session formats but remain inconsistent in magnitude and durability, particularly outside research settings [15, 19, 20]. Drawing on one of the most recent reviews, Madhombiro et al. [15] reported that only three of 21 studies among people with HIV showed significant reductions in drinking quantity or frequency. Specifically, these interventions emphasized a diversity of approaches including motivational interviewing (MI) [21], cognitive behavioral therapy (CBT) [22]; and education and building of skills [23]. Overall, these findings suggest that while success has been inconsistent, approaches that actively build skills and harness self-directed motivation have yielded the clearest signals. Furthermore, authors of reviews note that interventions that recruit patients from clinic settings tend to be more effective [18], and also suggested that alcohol use outcomes other than retrospective self-report, including biomarker data or ecological momentary assessment data [24], could help to increase the rigor of the studies [16]. Together, the literature highlights three persistent gaps/opportunities motivating the present work: the importance of interventions that combine an MI style to strengthen commitment with CBT-based skills training to manage triggers and high-risk situations; the need for scalable, remote/technology delivery models that sustain engagement; and the need to supplement self-report with continuous, ecologically valid monitoring [e.g., ecological momentary assessment (EMA) [24] and objective alcohol biomarkers] to both strengthen measurement and activate behavior-change processes [15–17].

Researchers have also sought to evaluate the effects of alcohol reduction interventions specifically among VWH, though the number of such studies is small. A chart review study of clinic-based brief interventions among VWH found no effect of interventions on resolution of unhealthy alcohol use [25]. Similarly, stepped care interventions that enrolled VWH having alcohol misuse and alcohol use disorder did not yield significant effects on primary alcohol use outcomes for these two populations, but were effective in reducing alcohol use for VWH having comorbid liver disease [26–28]. More recently, mobile/smartphone interventions in veteran samples have reported encouraging within-participant changes: Malte et al. [29] observed significant decreases over six months in heavy drinking days and alcohol-related problems, and Blonigen et al. documented pre-post declines in 30-day total drinks, drinks per drinking day, and percent heavy drinking days [30]. However, both studies lacked a control condition, limiting causal inference about intervention efficacy. Insights from these studies suggest that there is a need for controlled trials [29, 30], that more intensive interventions (other than single-session brief interventions) may be necessary for this population [25], and that remote approaches, which can address logistical challenges associated with participating in research and clinical care interventions, may help to increase individuals’ willingness to enroll in and complete an intervention [26].

Given these challenges with alcohol reduction interventions identified in prior literature, we developed a new intervention – TRAC (Tracking and Reducing Alcohol Consumption) – that: (1) Involves 8 brief intervention sessions delivered over an 8-week period to offer more intensive alcohol reduction support; (2) Was delivered remotely via mobile phone in order to increase scalability and improve accessibility; and (3) Used daily ecological momentary assessment and biomarker (i.e., breathalyzer) data in addition to self-report. The intervention replicates core elements of prior multi-session interventions: motivational enhancement (self-directed motivation, personalized feedback and goal setting), CBT-informed skills (functional analysis of triggers, urge/craving management, drink-refusal, coping under stress), and brief mindfulness exercises to support emotion regulation, delivered in an MI style [21–23]. TRAC extends this work in three ways: [1] fully remote delivery (phone/video) to increase reach and reduce logistical barriers; [2] twice-daily monitoring via mobile ecological momentary assessment and a smartphone-paired breathalyzer to augment self-report with objective data; and [3] data-informed coaching, in which the interventionist reviews monitoring trends at the start of each session to personalize feedback and reinforce skills in real time. Self-monitoring, a well-known behavior change technique theorized to be a necessary first step in regulation of one’s own behavior [31], has been associated with greater effect sizes when used alongside brief alcohol interventions [32], and was thus included to enhance the intervention while also providing daily alcohol use data.

The TRAC intervention was tested among a sample of 50 VWH through a randomized waitlist-controlled pilot trial. The goal of this pilot trial was to determine if it was appropriate to move forward with larger-scale efficacy testing of the intervention through a focus on feasibility and acceptability, while also piloting intervention procedures and content to prepare for a future randomized controlled trial. Additionally, preliminary efficacy analyses were conducted to help support future sample size calculations. Specifically, we explored the following aims:

1. Evaluate the feasibility of TRAC by examining intervention enrollment, intervention adherence (i.e., % completing at least 6/8 intervention sessions; benchmark ≥ 70%), daily data collection adherence (median % of daily surveys and breathalyzer readings completed; benchmark ≥ 70%), and intervention retention (% retained through follow-up; benchmark: ≥70%).
2. Evaluate the acceptability of TRAC by assessing average ratings of intervention sessions, attitudes toward session content, and perceived therapeutic alliance as collected through post-session self-reports.
3. Assess the preliminary efficacy of the intervention by examining effects on alcohol use frequency, alcohol quantity, binge drinking episodes, and hazardous alcohol use using both between- and within-group analyses.

If the TRAC intervention is shown to be feasible, acceptable, and preliminarily efficacious, it will represent the first stage in a line of research intended to produce a highly scalable, fully remote intervention that replicates proven MI- and CBT-based elements while extending prior multi-session work through twice-daily EMA and smartphone breathalyzer monitoring with data-informed coaching. By addressing key gaps such as access barriers, over-reliance on retrospective self-report, and the need for objective, ecologically valid feedback mechanisms, TRAC has the potential for addressing the significant challenge of alcohol misuse among VWH and advancing the literature of brief, remotely-delivered alcohol interventions.

## Methods

### Study overview

**Fig. 1:**
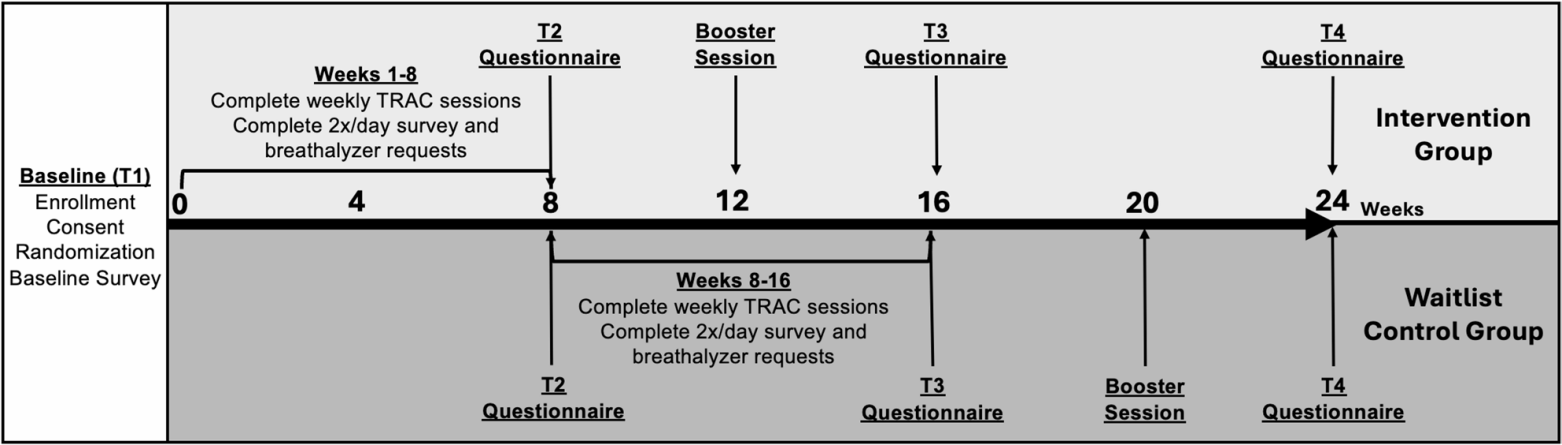
Participant timeline

We conducted a randomized waitlist-controlled pilot trial with 50 VWH who reported alcohol misuse. The target sample size of 50 was based on guidelines put forth for pilot studies of interventions and mirroring practice of similar pilot projects in the literature [33–35]. Participants were randomized at baseline via a computer-generated random number table either to an intervention or waitlist control group. Neither participants nor study staff were blinded to randomization conditions. A waitlist control condition was chosen after consultation with providers at the VA, who wanted to make the intervention available to all participants as quickly as possible given the potential negative impact of continued alcohol misuse among people who have HIV. While waitlist-control groups have noted disadvantages, such as inflated effect sizes [36, 37], they are common in alcohol reduction interventions [38–40] and in this case represented an approach that balanced scientific rigor with equity and principles of beneficence. The TRAC intervention includes 8 sessions plus a booster session delivered via videoconferencing and/or mobile phone and twice-daily monitoring of alcohol use through mobile surveys and breathalyzers. Participants randomized to the waitlist-control started the intervention at the 8-week timepoint. All participants completed questionnaires at baseline (T1), 8 weeks (T2), 16 weeks (T3), and 24 weeks (T4). For a visual depiction of participant flow for each of the study groups, see Fig. 1.

### Participants and recruitment

Participants for this study were recruited from the Infectious Diseases Clinic of the Atlanta Veterans Affairs Medical Center (VAMC). The Atlanta VAMC patient population is predominately male (84%) and over the age of 55 (56%). The largest demographic group for race at the VAMC is Black or African American (44%). Potential participants were pre-screened by VA research staff for recruitment to ensure that they met initial eligibility criteria related to age (18 and older), HIV status (HIV seropositive as documented in their chart), and prior alcohol use (must have any alcohol use codes in their medical record). Study coordinators would then call this list of potentially eligible patients to tell them about the study, which was described as “a study to help people with HIV reduce their alcohol use.” If individuals were interested, the study coordinator would then administer the study screener via phone.

The screener confirmed that participants met the following eligibility criteria: (1) HIV-seropositive as documented in their medical chart, (2) 18 years or older, (3) Currently taking ART, and (4) Engaging in alcohol misuse. For this study, alcohol misuse was defined as consuming more than three drinks (for women)/more than four drinks (for men) in a day or consuming more than 7 (for women)/14 (for men) drinks in a week over the past 3 months. These criteria were based on the National Institutes on Alcohol Abuse and Alcoholism’s definitions of at-risk drinking (NIAAA, https://www.niaaa.nih.gov/alcohols-effects-health/alcohol-drinking-patterns). Participants were excluded from the study if they had score of 20 or higher on the AUDIT questionnaire [41], which suggested alcohol dependence and likely required a more intensive approach than a remote intervention given the potential for alcohol withdrawal. These individuals were referred to their provider for treatment with Addiction Medicine specialists, with treatment offered according to what was deemed appropriate by their providers. Eligible and interested individuals were mailed an informed consent form, which the coordinator reviewed with them via phone call. Completed informed consents were then mailed back to the VA. All research procedures were approved by the Emory University Institutional Review Board and the Atlanta VA Research and Development Committee.

### TRAC intervention content

TRAC is a manualized intervention based on the principles of motivational enhancement, incorporating elements of cognitive behavioral therapy and mindfulness to support skill building and coping with urges. It is focused on increasing motivation to reduce drinking, setting alcohol reduction goals, identifying triggers for alcohol use, and generating strategies for addressing those triggers. It was delivered via 30–45 min videoconferencing or mobile phone sessions, conducted on a weekly basis over an 8-week period, and using an MI-based approach. Additionally, participants completed a short booster session 4 weeks after the intervention concluded (see Table 1 for a description of intervention session content). All participants were provided a paper workbook that they completed during conversations with the interventionist, which allowed them to keep track of their goals, triggers, and strategies. While this was a mobile health intervention, a paper workbook was needed so that participants could easily access it while talking on the phone with the interventionist. Each week, the interventionist would call the participant via phone or video according to their choice, confirm they had a copy of the participant manual to reference during the session, and began following the manualized intervention. While the intervention itself was structured, it contained many open-ended questions that encouraged the participants to reflect on their behavior, set goals for themselves, and engage in change talk. On the day prior to sessions, the interventionist would text the participants a reminder of their scheduled time.

**Table 1:** TRAC intervention content

Session Emphasis	Session Content
1. Study Introduction	Study Overview
	Self-monitoring/phone training
	Breathing exercise
2. Developing a Change Plan	Increasing motivation
	Developing a change plan
	Anticipating challenges to change plan
3. Triggers for Drinking Alcohol & Distraction Techniques	Identifying urges & cravings
	Identifying emotional triggers
	Identifying situational and environmental triggers
	Identifying social triggers
	Brainstorming distraction techniques
4. Skill Building: Managing Urges and Cravings	Delay before acting
	Negative consequences of drinking
	Positive consequences of not drinking
5. Skill Building: Managing Emotional Triggers	Improve the moment
	Do something relaxing
6. Skill Building: Managing Social Triggers	Drink refusal
	Seek social support
	Seek spiritual support
7. Skill Building: Managing Situational and Environmental Triggers	Having alternate food or drink
	Engaging in alternate behavior
	Avoiding the situation or environment
8. Looking Ahead	Summative self-assessment
	Identifying challenges to behavior maintenance
	Building confidence
Booster Session	Review of original goals
	Review progress toward goals
	Plans for addressing setbacks or challenges

In addition to these weekly sessions, participants also completed twice-daily monitoring of their alcohol use using mobile surveys (programmed with Qualtrics) and BACTrack Mobile breathalyzers. Automated text message software was used to randomly send participants a request to complete a survey and breathalyzer reading at some point before 5pm and again after 5pm. Individuals could set “do not disturb” hours for times in which they were typically unavailable due to work, education, childcare, or sleep. The text message contained a link to the mobile survey, which they completed in their phone’s web browser, and a reminder to complete and send in a breathalyzer reading using the BACTrack app. If participants did not complete the reading within 90 min, follow-up reminders were sent. Participants were instructed to complete a survey and breathalyzer reading before bed if they had not yet received their evening reminder in an effort to increase response rates. The interventionist reviewed the results of the mobile surveys and breathalyzers with the participants at the beginning of each session in relation to their alcohol reduction goals and their progress. If participants did not have access to a smartphone with reliable data, they were provided a study smartphone to use for the duration of the study. All participants were also mailed a BACTrack breathalyzer. During the first intervention session, the interventionist provided extensive training on how to use the phone and breathalyzer and ensured the participant was able to successfully complete a breathalyzer reading. The participant workbook also contained visual instructions of how to complete surveys and breathalyzer readings that participants could reference whenever needed.

### Participant compensation

Participants received several types of stipends throughout the intervention to support their participation. They received $20 in incentives for attending each session and up to $15 compensation each week for completing daily monitoring tasks ($1 for each completed survey/breathalyzer and a bonus $1 for completing all readings). They also received compensation of $30 for the baseline questionnaire and $40 for follow-up questionnaires. Overall, the total amount of stipends they could receive was $450 across the 24 weeks of participation. The provision of stipends for attending intervention sessions was in response to previous studies demonstrating the effectiveness of monetary rewards for increasing attendance at counseling sessions and medical appointments among individuals with substance use disorders and those with HIV [42–45], though there are drawbacks to this approach (see Limitations section). Stipends provided for daily monitoring are similar to those used in previous studies of interventions that incorporated a daily monitoring component [46–48].

## Interventionist training

The interventionist for this study was an individual with a master’s degree in public health who had received advanced training in MI. The principal investigator trained the interventionist on the TRAC manual through a high-level overview and detail on the principles underlying the manual. The interventionist conducted several mock sessions observed by the principal investigator until it was determined that the intervention was being delivered as intended. A small open trial with 10 participants, previously published [49], was conducted to further train the interventionist, determine areas needed for improvement, and ensure the intervention content was being delivered successfully. Feedback from participants on the interventionist and the content itself was extremely positive, demonstrating readiness to complete the pilot randomized trial. For privacy reasons related to protecting patient data at the VA, we were not able to record the sessions to determine intervention fidelity. To provide insight as to whether or not the interventionist was adhering to MI principles, participants were invited to complete a measure of therapeutic alliance after each session (see *Measures* section), which has been shown to be correlated with the use of MI skills [50, 51].

### Measures

After every intervention session, participants were invited to complete a short questionnaire that asked them to rate the session on a scale of 1–10. They also completed several 5-point semantic differential attitudinal measures that ranged from − 2 to 2 rating the session, including Boring/Interesting, Unpleasant/Pleasant, Bad/Good, Useless/Useful, and Negative/Positive. Scores on the session attitude semantic differential measures were reverse-coded where required and averaged to form an overall attitude score, with “0” being “neutral.” Finally, they completed the Working Alliance Inventory- Short Revised [52], which contains 12 items and assesses agreement between the participant and the interventionist on intervention tasks, goals, and the development of an affective bond. Scores were averaged for each survey response to form an overall score of perceived therapeutic alliance.

As part of baseline and follow-up measures, participants completed the AUDIT-C, a widely-validated measure to assess hazardous alcohol use [53]. Additionally, for the past 30 days, participants reported number of drinking days, average number of drinks/drinking day, and number of binge drinking episodes (4 or more for women on one occasion, 5 or more for men), based on questions from the Behavioral Risk Factors Surveillance System [54]. They also reported how many doses of their ART they had missed in the previous 7 days using previously-validated questions recommended for use in assessing adherence [55]. Finally, they completed a treatment self-efficacy scale to assess their confidence in sticking to their HIV treatment plan, which is meant to encompass both ART adherence and other treatment-related behaviors (e.g., nutrition, exercise) and has been validated in prior research [56].

For daily monitoring, measures analyzed in this paper included morning survey questions asking participants if they had taken all of their prescribed ART on the previous day (yes/no) and if they had consumed any alcohol on the previous day (yes/no). They also completed a breathalyzer reading at the time of the morning survey, and another breathalyzer reading in the evening. Breathalyzer results that were above 0.0 were labeled as “positive,” while values of 0.0 were “negative.”

### Data analysis

#### Aim 1 and Aim 2 analyses

We used descriptive summary statistics (e.g., means, standard deviations, counts, proportions, IQR) to assess feasibility metrics, including intervention session ratings, enrollment rates, intervention adherence (defined as the percentage of participants completing at least 6 of 8 sessions), daily data collection adherence (median % of completed daily surveys and breathalyzer readings), and retention rates at follow-up. Thresholds for feasibility were defined a priori (e.g., ≥ 70% adherence and retention) based on our preliminary studies and prior research [20, 49, 57]. Additionally, to assess if alcohol use impacted individuals’ ability to adhere to monitoring tasks, we conducted a Spearman correlation analysis to assess the relationship between overall adherence to breathalyzer readings/daily surveys and past-30 day alcohol use (number of drinking days or average number of drinks) as reported at the assessment immediately following the monitoring period (T2 for the intervention group, T3 for the waitlist-control).

### Aim 3 analyses

We conducted descriptive statistics for the outcome variables, including mean and standard deviation for continuous variables and counts and percentages for categorical variables, as well as standardized mean differences, and summarized them across the intervention groups. We conducted within-group analyses to examine pre- to post-intervention changes in alcohol-related outcomes, including frequency of alcohol use, quantity consumed, number of binge episodes, and AUDIT-C scores. Paired-sample t-tests and or Wilcoxon signed-rank tests (for non-normally distributed data) were used to assess changes. Effect sizes (Cohen’s d) were calculated to inform the magnitude of change and to guide future sample size calculations for a fully powered trial (Tables 3, 4 and 5). In assessing the differences in alcohol use and ART adherence between the two groups during the 8-week intervention period, we utilized the Kruskal-Wallis test, a non-parametric statistical approach, to confirm the distribution of outcome variables. The general linear model (ANOVA) and two-sample matched t-tests (Welch test) were used to estimate the difference in differences between alcohol use and ART adherence among the groups, and the estimation of the effect size (Cohen’s D) at the end of the 8-week intervention period. The difference-in-differences approach attempts to estimate the causal effect of an intervention by comparing changes from baseline to follow-up between the treatment and control groups. While this study was not powered to detect significant differences, the goal of these analyses was to observe changes and effect sizes that may provide preliminary evidence of efficacy.

To examine intervention effects as assessed through daily surveys and breathalyzers, responses to the morning survey question asking if participants had consumed alcohol on the previous day (yes/no) and whether or not breathalyzer results that were returned were positive or negative were examined. For the first week of the intervention period and the last week of the intervention period, the change in the proportion of participants’ daily alcohol use (i.e., % of days drank during the first week compared to the % of days drank during the final week) and the proportion of positive breathalyzer readings were assessed. Pre-post analyses were completed because participants did not complete daily monitoring during the waitlist period; thus, we could not compare daily monitoring results between the intervention and waitlist control condition. Two methods were used: first, we used a one-sided paired samples t-test to assess reductions in the proportion of drinking days and positive breathalyzer readings while controlling for repeated measurements at the individual level. Second, a McNemar’s matched pair test was used to assess changes in the proportion of drinking days and positive breathalyzer readings across all participants. We also conducted a Spearman correlation analysis to assess concordance between the proportion of self-reported drinking days and positive breathalyzer readings at both Week 1 and Week 8 time points. Missing data were treated as missing at random and remained missing in all analyses. Effects on medication adherence using daily data could not be assessed due to a lack of variance in the responses to the question regarding whether or not participants had taken their HIV medication (participants responded “yes” to taking their medication 99% of the time).

All participants received the intervention by the end of the T3 assessment (16 weeks post-baseline). To examine long-term intervention effects, participant data from the active treatment interval from both the waitlist-control and intervention conditions were combined into a single dataset. A generalized linear model (via SAS GENMOD PROCEDURE) was fit to the change from baseline for each outcome measure: the number of days when a participant consumed alcohol in the previous 30 days, average number of drinks per drinking day, number of binge drinking episodes, and AUDIT-C scores at each time point. Change from baseline was calculated as the difference between the baseline value and values obtained at subsequent time points: 8 weeks (immediate post-intervention; T2 for intervention group and T3 for waitlist-control) and 16 weeks (2 months post-intervention; T3 for intervention group and T4 for waitlist-control) for each outcome measure. The rationale for fitting the change from baseline is that this model removes the effect of baseline differences that may exist from the model. All statistical hypothesis tests were conducted used the standard 5% significance level. SAS Version 9.4 (TS1M1, SAS Institute, Cary, NC), was used for all analyses.

## Results

### Participant demographics

**Fig. 2:**
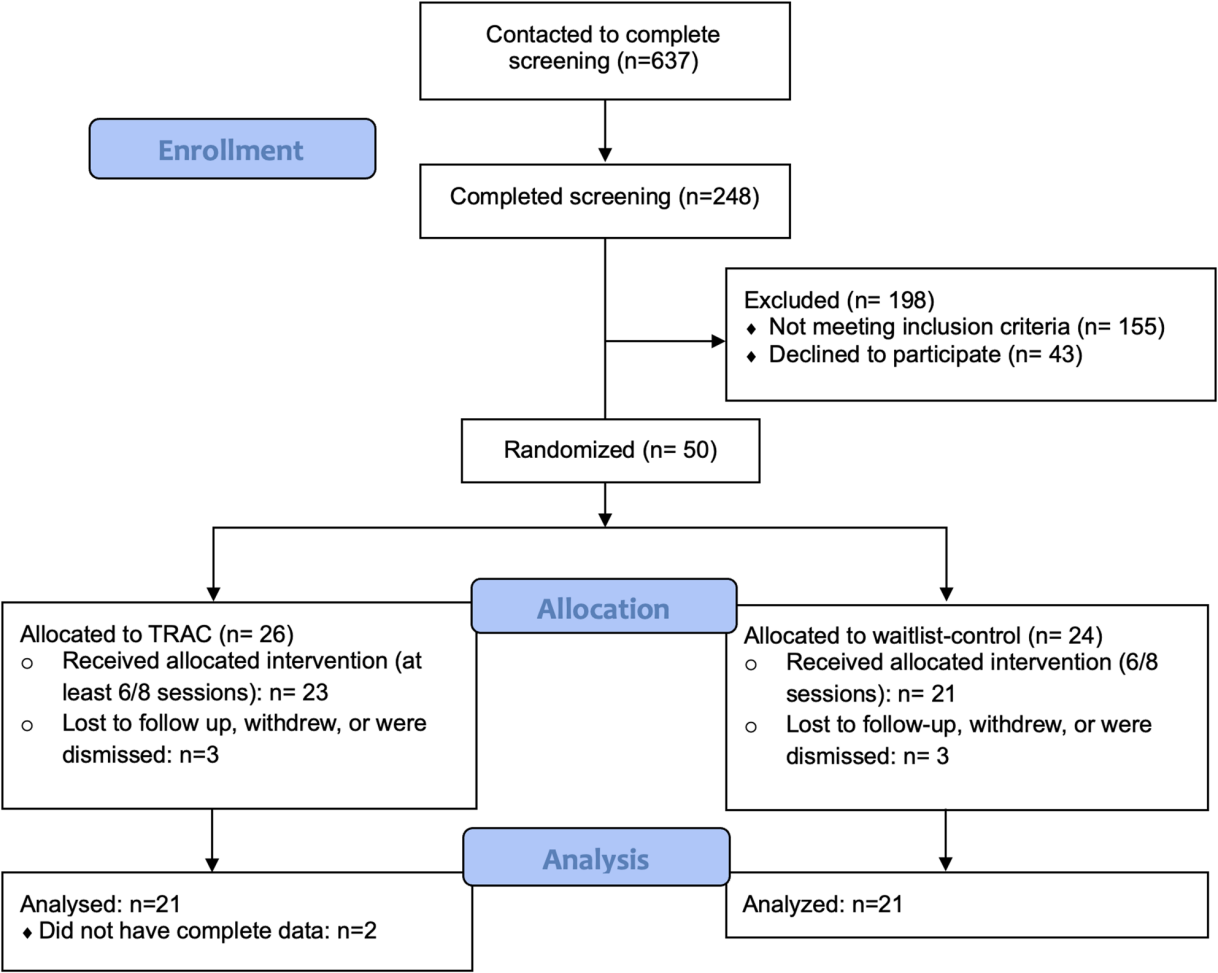
TRAC CONSORT diagram

See Table 2 for detailed participant demographics as collected at baseline. Overall, the majority of participants were male-identifying, Black/African-American, and reported being gay or bisexual. They had an average age of 53 years and most were not currently working. Participants were recruited into the study between April 2020 and January 2023, with accrual showing little seasonal variation across the study (11 accrued in Spring, 13 in Summer, 15 in Fall, and 11 in Winter). See Fig. 2 for the study CONSORT diagram.

**Table 2:** Participant demographics (*N* = 50)

Demographic	*N* (%) or M(SD)
**Average Age**	M = 53.34 (SD = 10.95)
**Sexual Orientation**	
Gay	24 (48%)
Bisexual	10 (20%)
Straight/Heterosexual	14 (28%)
Other sexual orientation	2 (4%)
**Gender Identity**	
Male	48 (96%)
Female	1 (2%)
Nonbinary	1 (2%)
**Hispanic/Latino Origin**	
No	47 (94%)
Yes	3 (6%)
**Race**	
Black/African American	46 (92%)
Multiracial	2 (4%)
White	2 (4%)
**Education**	
High school or GED	9 (18%)
Some college	20 (40%)
College graduate	9 (18%)
Some graduate or professional school	4 (8%)
Completed graduate or professional school	8 (16%)
**Personal Income**	
$0–9,999	12 (24%)
$10,000–19,999	15 (30%)
$20,000–34,999	9 (18%)
$35,000–49,999	3 (6%)
$50,000–74,999	6 (12%)
$75,000 or more	2 (4%)
**Employment Status**	
Working full-time	11 (22%)
Working part-time	3 (6%)
Not working	36 (72%)
**Average Time Since Diagnosis**	21.17 years (10.63)

### Aim 1: intervention feasibility

Over 600 participants were recruited to participate in the study, with 39% (248/637) completing the screening questionnaire to determine eligibility. Of the 93 individuals who were eligible for the study, 50 participants enrolled and completed the baseline assessment (54% enrollment rate). Of the 50 who were enrolled, 42 completed all follow-up assessments (84% retention rate). 42 participants completed all 8 intervention sessions plus the booster (84%), with 44 completing at least 6 sessions (88%). Three participants withdrew before completing any intervention sessions, with another three completing 2 or fewer sessions.

The median percentage of mobile surveys completed across the 8 weeks of the intervention was 85% (*M =* 71.70%, *SD =* 29.76, *IQR =* 44.50), and the median percentage of breathalyzer readings completed was 78% (*M =* 66.13%, *SD* = 26.29, *IQR* = 43.50). There were no significant correlations between adherence to breathalyzer readings or daily surveys and past-30 day alcohol use (number of drinking days or average number of drinks) at the assessment immediately following the monitoring period (T2 for the intervention group, T3 for the waitlist-control), suggesting that participants’ alcohol use did not impact their ability to adhere to monitoring protocols.

### Aim 2: intervention acceptability

The average rating for intervention sessions was 9.60 (SD = 1.07, IQR = 0.00) out of 10. The average attitude score toward session content was 1.01 (SD = 1.55, IQR = 2.00), with 0 representing “neutral” and 2 representing the most favorable attitude score possible. Average perceived therapeutic alliance was 4.44 (maximum score of 5; SD = 0.72, IQR = 0.83).

### Aim 3: preliminary efficacy findings

#### Intervention effects vs. Waitlist control

Preliminary efficacy analyses were conducted to compare participants’ alcohol use, ART adherence, and treatment self-efficacy in the intervention group with those in the waitlist control group during the initial 8-week intervention period. The results indicate that there were differences in reductions in alcohol use and medication-related outcomes between the two groups, but they were not statistically significant at the 5% significance level, likely due to low power resulting from the small sample. The largest effect size for alcohol-related outcomes was for binge drinking (D = 0.16), with the intervention group reporting a decrease of 3.00 past-month binge drinking episodes compared to a decrease of 1.89 episodes in the control group. Regarding medication outcomes, there was a small effect size (D= -0.21) for missed medication doses, with the intervention group reporting 0.24 fewer missed medication doses compared to no change in the control group. Additional outcomes are listed in Table 3.

**Table 3:** Comparison of the intervention and Waitlist-Control groups during the intervention period

	Intervention Group				Control Group								
Measure	N	T1 Mean (SD)	T2 Mean (SD)	Mean Diff (SD)	N	T1 Mean (SD)	T2 Mean (SD)	Mean Diff (SD)	Diff of Diff	P-Value	Cohen’s D	95%CI	SMD
Drinking Days	21	13.86 (8.88)	10.77 (8.41)	-3.09 (7.61)	20	17.10 (9.71)	15.00 (9.97)	-2.10 (12.48)	-0.99	0.762	-0.10	(-0.72, 0.53)	-0.22
Average number of drinks	18	3.44 (2.18)	2.67 (1.88)	-0.78 (2.07)	17	3.24 (1.79)	2.74 (1.70)	-0.50 (2.42)	-0.28	0.719	-0.12	(-0.79, 0.55)	0.19
Binge drinking episodes	20	5.35 (8.60)	2.35 (3.05)	-3.00 (7.06)	19	5.79 (7.81)	3.89 (6.97)	-1.89 (6.50)	-1.11	0.614	-0.16	(-0.83, 0.51)	0.08
AUDIT-C	21	4.62 (2.04)	3.67 (2.27)	-0.95 (1.50)	20	5.40 (2.82)	4.35 (2.72)	-1.05 (3.32)	0.10	0.905	0.04	(-0.59, 0.66)	-0.11
Missed Medication Doses	21	0.90 (1.61)	0.67 (0.91)	-0.24 (1.45)	21	0.43 (0.68)	0.43 (0.68)	0.00 (0.78)	-0.24	0.511	-0.21	(-0.84, 0.41)	0.08
Treatment Self Efficacy	21	94.81 (19.50)	103.57 (19.49)	8.76 (18.63)	21	102.71 (17.7)	109.33 (10.8)	6.62 (12.11)	2.14	0.661	0.14	(-0.48, 0.75)	0.06

Note: SMD: Standardized mean difference at baseline (T1; intervention – control)

### Daily monitoring outcomes

Analyses of daily drinking data at the individual level via paired samples t-tests demonstrated a non-significant reduction in the proportion of drinking days at Week 1 (43.16%, SD = 0.39) compared to Week 8 (38.86%, SD = 0.39), *t =* 0.65, one-sided *p =* 0.26. The McNemar’s matched-pair test assessing change across all participants indicated a decrease in alcohol use from an average of 44.06% (89 out of 202) of days drank during week 1 to 39.50% (79 out of 200) of days drank during week 8 (χ^2^ = 4.88, p -value = 0.027). Consequently, the odds of using alcohol during week 8 were estimated to be 0.83 (95% CI: 0.56, 1.23) compared to week 1, suggesting participants were 1.20 times less likely to report drinking days during week 8.

Analyses of breathalyzer results at the individual level via paired samples t-test demonstrated that the average proportion of positive breathalyzer readings was 33.18% (SD = 0.31) at week 1 and 24.82% (SD = 0.29) at week 8 (*t* = 1.93, one sided *p* = 0.03). In examining the distribution of the number of positive breathalyzer readings across all participants at Week 1 vs. Week 8, we observed a 4.31% reduction from 29.57% (118 out of 399) at week 1 to 25.26% (96 out of 380) during week 8 (χ^2^ = 68.50, *p* < 0.001). Subsequently, the odds of observing a positive breathalyzer at week 8 were 0.81 (95% CI: 0.58, 1.10), indicating participants were 1.25 times less likely to produce positive breathalyzers during week 8.

Finally, to assess concurrency between self-reports of alcohol use and breathalyzer results, we examined correlations between number of drinking days and number of positive breathalyzer readings for Week 1 and for Week 8. For both Week 1 and Week 8, the number of drinking days was significantly correlated with the number of positive breathalyzer readings (Week 1: Spearman’s ρ = 0.58, *p* < 0.0001; Week 8: Spearman’s ρ = 0.61, *p* < 0.0001).

### Long-term outcomes

**Fig. 3:**
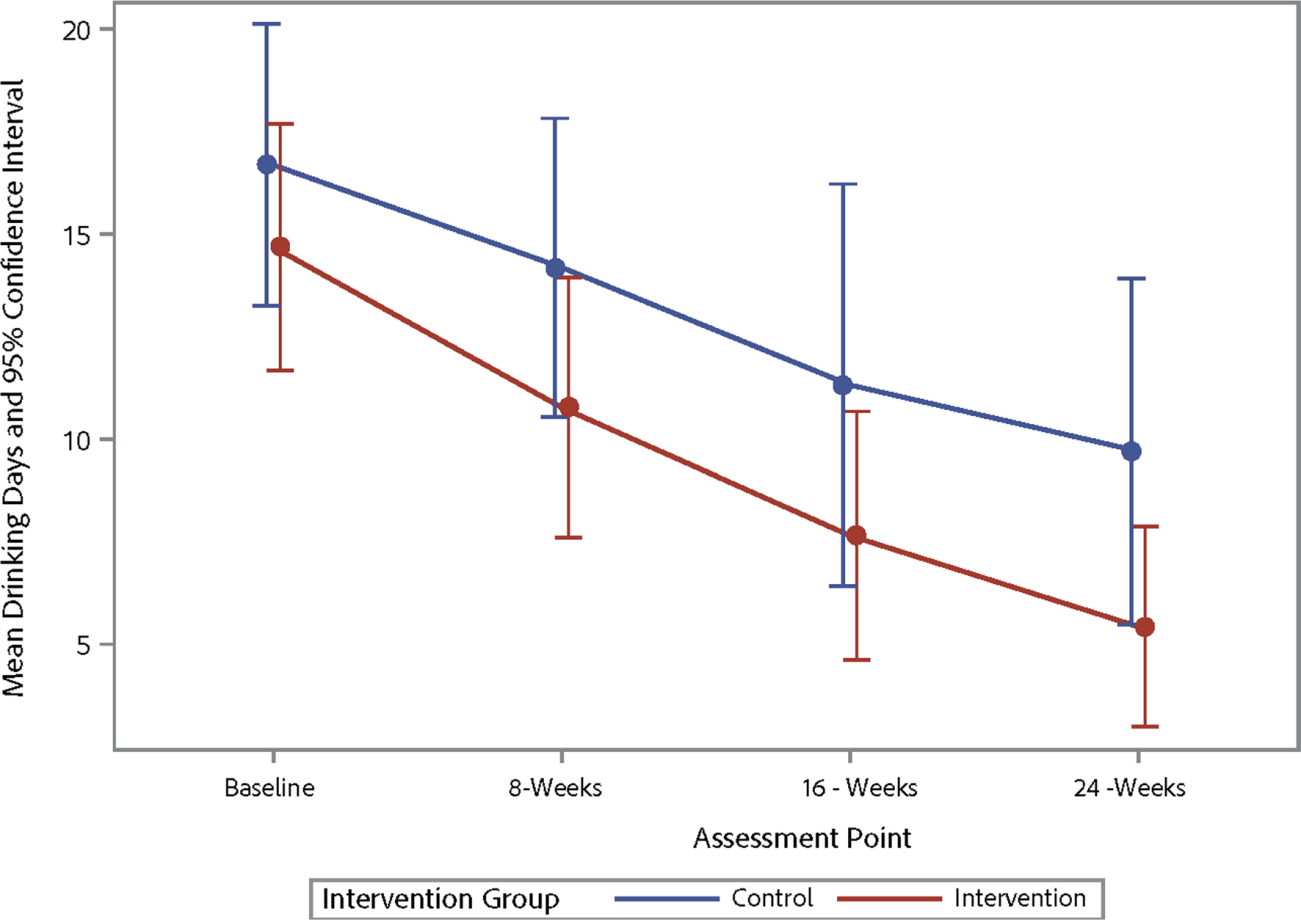
Changes in average number of drinking days by study group

**Fig. 4:**
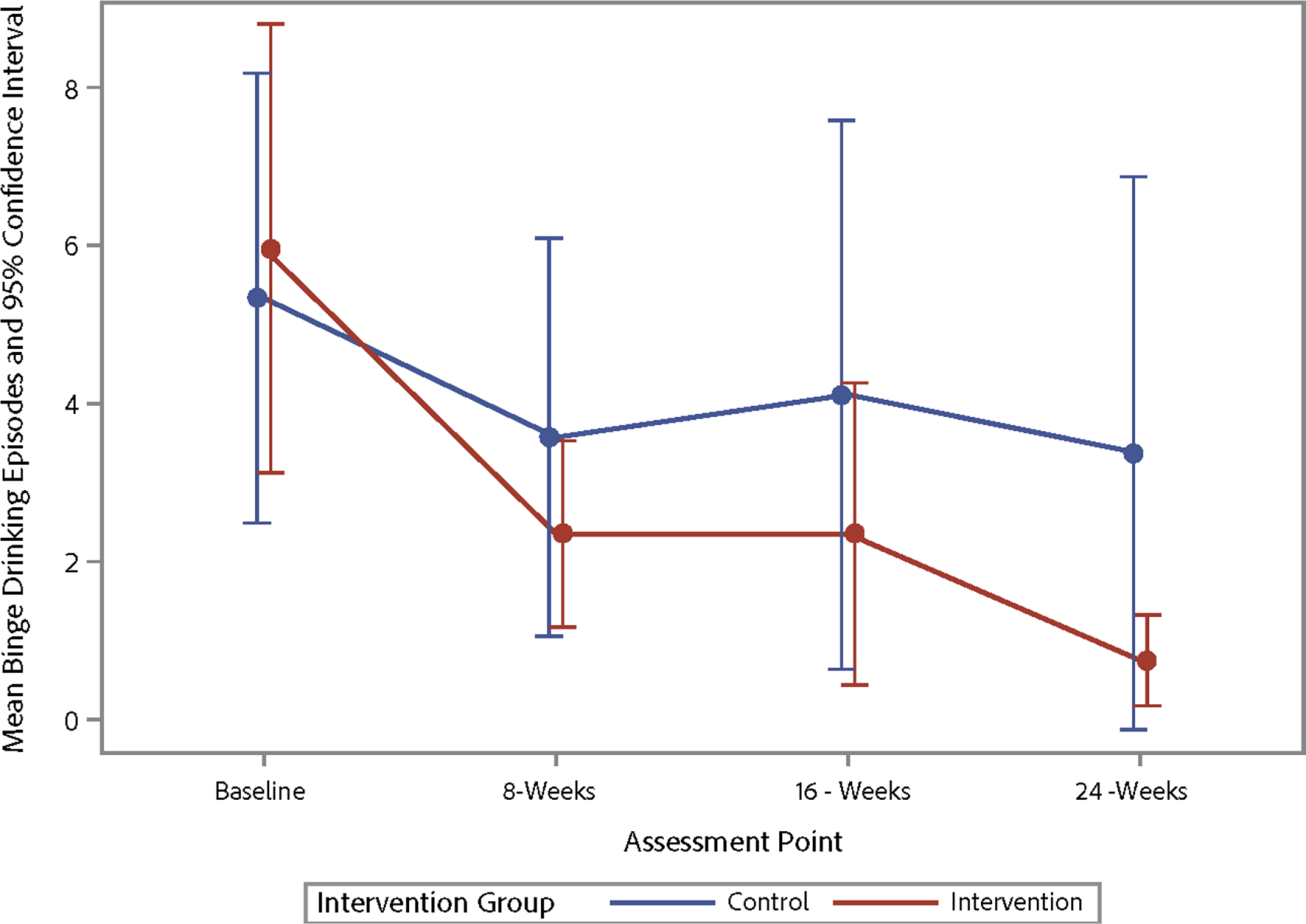
Changes in binge drinking episodes by study group

**Fig. 5:**
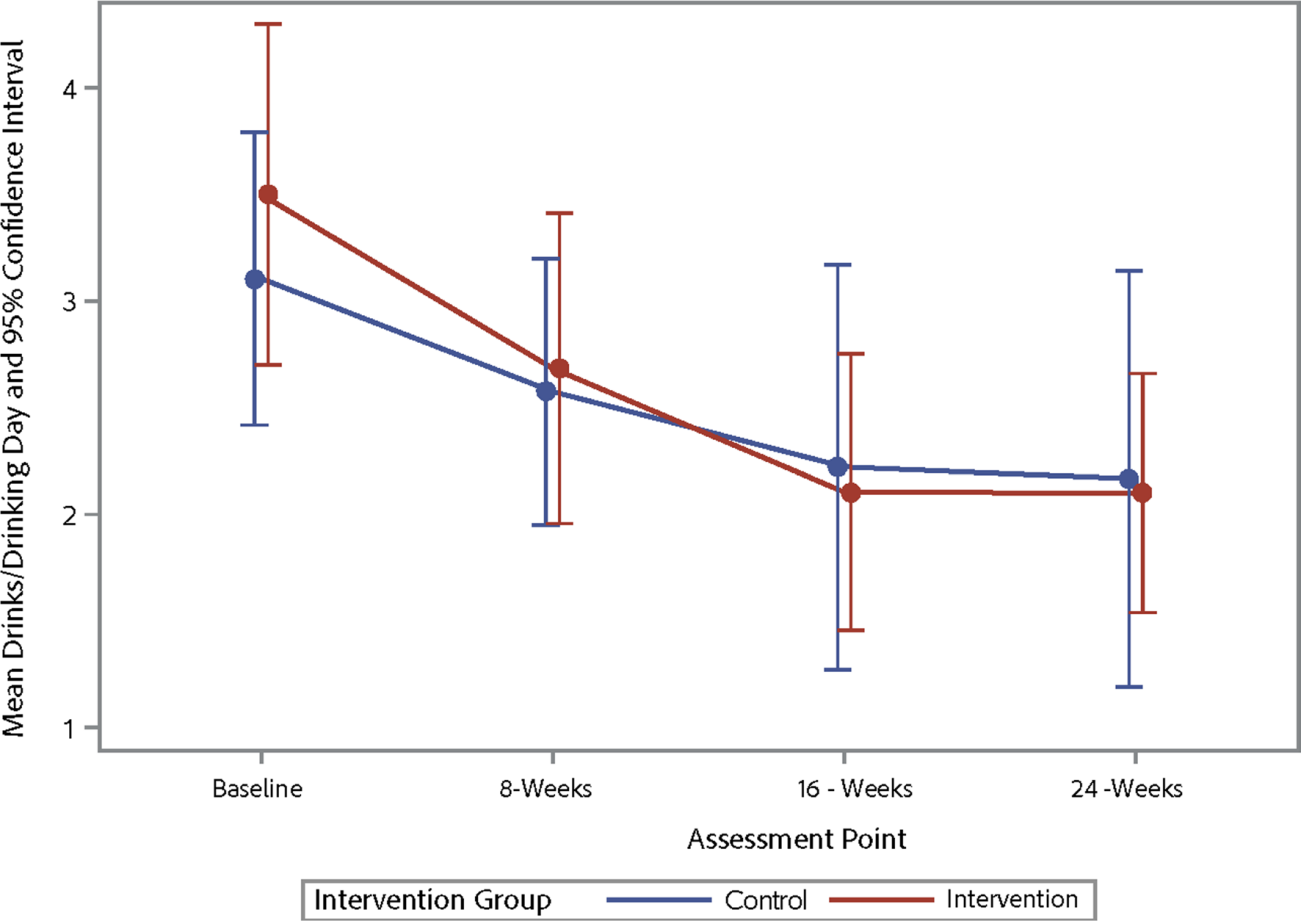
Changes in average drinks/drinking day by study group

**Fig. 6:**
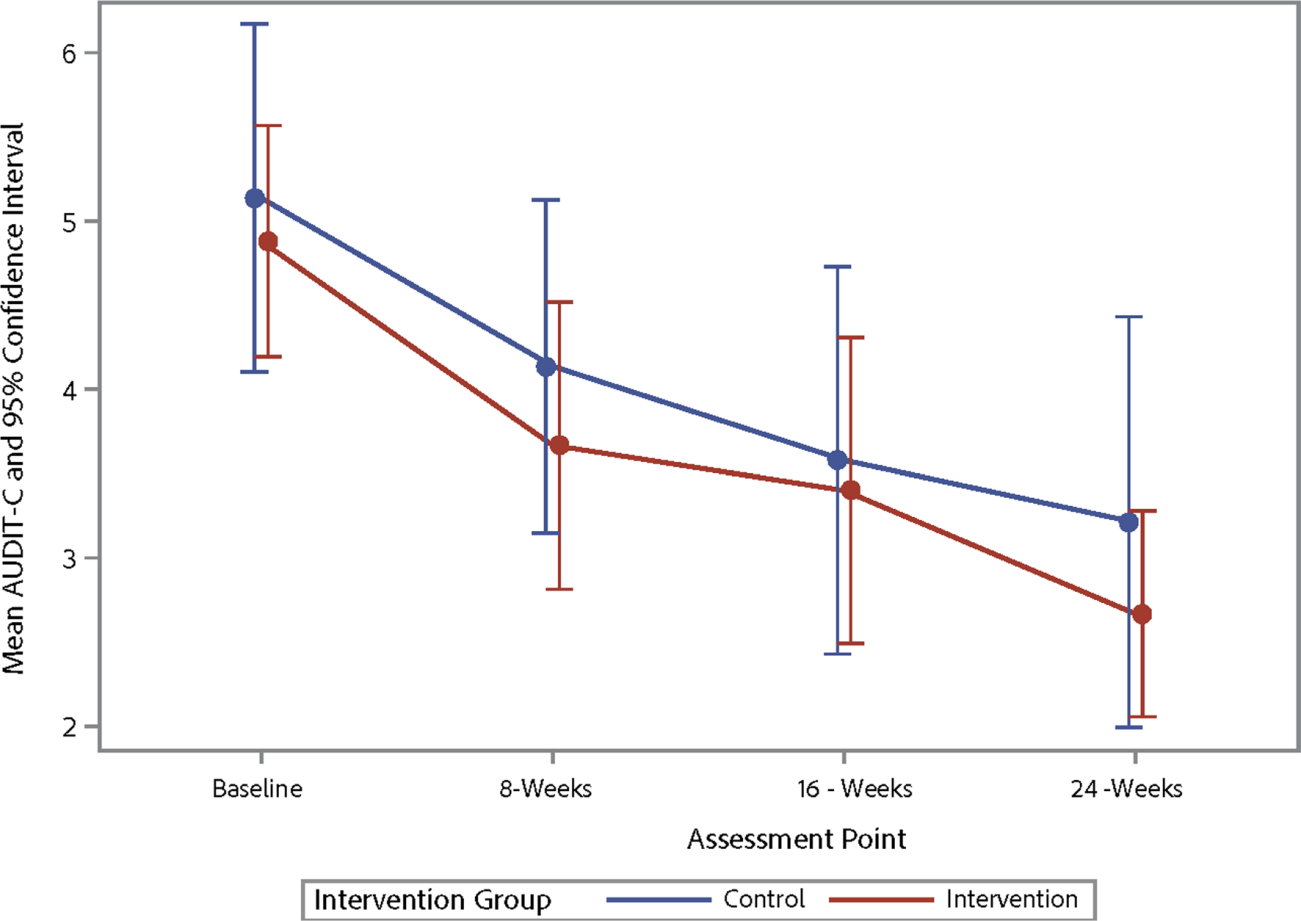
Changes in AUDIT-C scores by study group

Table 4 shows the changes from baseline to 16 weeks post-baseline for alcohol use and ART adherence outcomes with participants from the intervention and waitlist-control pooled into one sample, representing a pre-post comparison. The results indicate improvements in various alcohol use parameters, including the average number of days a participant drank alcohol, binge drinking episodes, and AUDIT-C scores. However, there were no significant changes in ART adherence. Table 5 shows the trajectory of changes in alcohol use and ART adherence across three-time points for the outcome variables. Figures 3, 4, 5 and 6 demonstrate the trajectory of alcohol use outcomes from baseline to the final post-intervention assessment.

**Table 4:** Change from baseline to 2-months post-intervention for alcohol use and medication adherence outcomes (*n* = 42)

Variables	Estimated Mean Change (SD)	Test Statistic	*P*-value
**Alcohol use**			
Drinking days	-5.8(10.44)	-3.50	0.001
Drinks/drinking day	-0.8(10.08)	-0.47	0.642
Binge drinking episodes	-2.2(5.43)	-2.54	0.015
AUDIT-C	-1.2(2.20)	-3.35	0.002
**Medication Adherence**			
Missed Doses	0.3(1.35)	1.39	0.172
Treatment Self Efficacy	1.8(18.52)	0.62	0.536

**Table 5:** Trajectory of changes across timepoints for key outcomes for pooled sample

	Baseline		Immediate Post Intervention		2 Months Post-Intervention	
	*N*	Mean (SD)	*N*	Mean (SD)	*N*	Mean (SD)
Drinking Days	47	14.45 (9.24)	40	11.03 (10.31)	40	8.68 (9.43)
Drinks/drinking day	44	3.08 (2.04)	37	2.46 (2.06)	37	2.14 (2.00)
Binge Drinking episodes	46	4.87 (7.62)	38	3.18 (6.21)	39	2.85 (7.01)
AUDIT-C	47	4.53 (2.36)	40	3.63 (2.55)	39	3.31 (2.69)
Number of Missed Doses	47	0.64 (1.12)	40	0.55 (0.88)	41	0.98 (1.57)
Treatment Self Efficacy	46	103.22 (16.92)	40	107.05 (16.08)	41	103.83 (19.73)

## Discussion

The primary goal of this pilot randomized waitlist-controlled trial was to establish feasibility and acceptability of the TRAC intervention. Results suggest that the intervention was indeed feasible to deliver and acceptable to participants, given high retention rates, high ratings of the intervention sessions, and high adherence to monitoring tasks. We previously conducted an open trial of this intervention with 10 participants that incorporated qualitative feedback, in which a strong majority reported that the TRAC technology was easy to use, that the remote delivery of the intervention increased convenience, that the intervention made participants more aware of their drinking habits and helped to change their behavior, and that they would recommend the intervention to others [49]. Other investigators have tested remote interventions to address health behaviors among people with HIV, including medication adherence, and have found similar results in terms of feasibility and acceptability [30, 58]. Given the stigma many people with HIV perceive or report experiencing in healthcare settings [59, 60], remote interventions such as the TRAC study may help to increase engagement and address gaps in the care continuum.

In examining alcohol use outcomes during the initial 8-week period, there were trends observed that suggested alcohol use decreased in the intervention condition relative to the control condition. As a pilot feasibility trial, this study was not powered to detect significant effects, so the lack of statistically significant findings was not surprising and effect sizes should be interpreted with caution. However, the trends observed were encouraging, especially in regard to decreases in binge drinking episodes. Interestingly, both the intervention and control conditions decreased their alcohol use during the initial 8-week period across multiple metrics. This has been observed in several prior studies of alcohol interventions for people with HIV [16, 61–63]. The reasoning behind this is unclear, as participants in the waitlist-control group did not receive any intervention materials and did not discuss alcohol use beyond completing the baseline questionnaire and consent form. It may be the case that simply qualifying for an alcohol reduction study, completing the baseline questionnaire and reflecting on their alcohol use, and/or knowing that they were going to report on their alcohol use again at a later date was enough to motivate participants to reduce their drinking even before starting the intervention. Alternatively, individuals who elected to join the study may have already been contemplating reducing their alcohol use. Importantly, the downward trajectory of alcohol use that waitlist-control participants initiated during the waitlist period for the large part continued after they began the intervention.

After pooling the intervention and control conditions in order to examine long-term effects, there were strong effects observed on alcohol use. Specifically, there were reductions in the number of drinking days, binge drinking episodes, and AUDIT-C scores from baseline to 2 months post-intervention. These findings suggest that the TRAC intervention was able to produce and sustain healthier drinking behaviors over time among VWH by reducing drinking days and binge drinking episodes; though, again, these results should be interpreted with caution given that they represent pre- post-outcomes (not intervention vs. control) in a relatively small sample. This finding aligns with that of previous meta-analyses that highlight the durable benefits of MI-based interventions on reducing substance use [64]. Furthermore, the reduction in AUDIT-C scores implies that hazardous drinking patterns, which adversely affect HIV treatment outcomes, may be mitigated with the TRAC intervention. Notably, post-test reductions in hazardous drinking are consistent with MI-oriented intervention trials reporting meaningful decreases [21, 65], including effect sizes in the moderate-to-large range, which supports the plausibility of TRAC’s MI/CBT ingredients as active contributors.

The between-and within-group effects observed on alcohol use outcomes, while very preliminary in nature, align with mixed but encouraging evidence that multi-session behavioral interventions can reduce alcohol use among people with HIV, albeit with variability in magnitude and durability [13, 15]. Among VWH, stepped-care and alcohol-care management models have generally shown limited effects on primary alcohol outcomes overall, with clearer benefit for some subgroups [17, 19]. Meanwhile, technology-delivered brief interventions in VA settings have produced heterogeneous or short-lived changes [66, 67], and recent mobile/smartphone evaluations report encouraging pre–post decreases yet typically lack concurrent controls [29, 30]. Against that landscape, TRAC targets recurring gaps related to access barriers and reliance on retrospective self-report, while preserving active ingredients linked to positive outcomes in previously effective trials (motivational enhancement and CBT-based skills training). The anticipated advantages over existing approaches are: (i) greater reach and engagement through phone/video delivery; (ii) improved measurement rigor that pairs self-report with EMA/breathalyzer readings to collect more ecologically valid data and simultaneously inform tailored feedback during intervention sessions; and (iii) closer alignment with behavior-change mechanisms (salience, feedback-guided self-regulation, self-efficacy) that can translate motivation into day-to-day reductions in drinking.

In examining effects on daily alcohol use while participants received the intervention, we observed a decrease in the proportion of positive breathalyzer readings at the individual level when comparing Week 1 of the intervention to Week 8. These findings provide further evidence that the intervention is able to affect meaningful change within an 8-week period and also point to the value of obtaining daily data in order to obtain more nuanced alcohol use outcomes. At the same time, as Taylor et al. [49] observed, without a monitoring-only control it can be difficult to disentangle counseling effects from self-monitoring reactivity. However, monitoring-only effects may attenuate within about a week [68], whereas TRAC’s reductions persisted across the 8-week active phase and at 2-months post-intervention, a finding that is more consistent with counseling plus skills practice rather than monitoring alone. Overall, the consistent reduction in alcohol use across assessment periods and outcomes underscores the value of further study of the TRAC intervention through a larger randomized controlled trial.

Regarding medication adherence outcomes, there was slight improvement for both missed ART doses and treatment self-efficacy, though these were not statistically significant. Additionally, pooled results showed that the TRAC intervention led to short-term improvements on ART adherence and treatment self-efficacy relative to baseline, but the effects were attenuated over time. Several reasons may account for this. First, because there was not enough variance in ART adherence to complete analyses with daily monitoring data, we were unable to derive a more nuanced and robust measure of change in adherence from baseline. Second, and more importantly, suboptimal ART adherence was not employed as a criterion for eligibility, and participants in the TRAC intervention were already predisposed to performing well on ART adherence (pooled sample mean of 91% adherence at baseline). The finding in a previous meta-analysis of ART adherence that stronger effects were more common in trials that required suboptimal adherence as an eligibility criterion [69] suggests that the efficacy of TRAC in improving medication use might be significant and greater were it focused on participants with the more serious adherence issues.

### Limitations

This pilot study, while promising, has important limitations that temper inferences and generalizability. First, efficacy conclusions are limited by design. Randomization created a short-term, between-group comparison during the initial 8-week period, but both arms received the intervention thereafter; consequently, longer-term (pooled) improvements cannot be causally attributed to TRAC and may partly reflect natural decline, assessment reactivity, or external influences. Although a waitlist-control design cannot isolate longer-term effects after crossover, it does provide more rigorous, albeit formative evidence of short-term efficacy than uncontrolled pre-post analyses by enabling a randomized, contemporaneous comparison during the initial 8-week period. As a pilot, the study was not powered to detect small effect sizes, so some non-significant findings may still be compatible with clinically meaningful difference.

Second, the use of self-reported data for alcohol use, despite the inclusion of breathalyzer readings, may introduce reporting bias. Participants might underreport their alcohol consumption or avoid submitting accurate breathalyzer readings while drinking due to social desirability or recall biases. Our breathalyzer analyses dichotomized 0.0 versus > 0.0, which may not capture low-level drinking or timing issues. Third, sample composition constrains external validity. Participants were predominantly men, largely Black/African American, from a single metropolitan VA in the U.S. Southeast, and mostly not working. While this reflects an important segment of VWH served at the VA, it limits generalizability to women, rural populations, other regions, and those with different socioeconomic profiles. We also excluded individuals with AUDIT ≥ 20, so results may not extend to veterans with alcohol dependence who likely need more intensive care.

Fourth, intervention fidelity was not formally assessed. Although delivery followed a structured manual and the interventionist received training on said manual, we did not collect structured fidelity ratings, precluding conclusions about the consistency and quality of delivery across sessions. While results related to perceived therapeutic alliance indicate that the interventionist was likely following principles of MI, this is not a substitute for systematic fidelity assessment. Finally, participants were paid to attend intervention sessions and complete daily monitoring, which may not be a scalable approach in a larger implementation of this intervention and may have inflated intervention adherence outcomes.

### Future research and implications

This pilot study represents an early stage in a line of research that will next seek to rigorously examine the efficacy of the TRAC intervention among people with HIV through a fully-powered randomized controlled trial, followed by a test of its effectiveness and ability to be implemented within clinical settings. If shown to be effective, TRAC has potential to be integrated into existing VA services, as the VA has long been on the forefront of telehealth and technology-delivered healthcare [70–72], with notable uptake of these services during the COVID-19 pandemic [73, 74]. The VA routinely assesses veterans for alcohol misuse using the AUDIT-C [75, 76], so TRAC could represent a referral pathway for patients who screen positive. Given the many facilitators the VA has in place for providing remote healthcare and their history of providing training to their clinical staff in MI [77, 78], there would likely be few barriers to deploying the intervention to their patients. This could potentially extend alcohol misuse treatment to the many VWH who live far away from VA clinical sites by reducing travel burden [79, 80]. If TRAC is shown to be effective in an RCT, a follow-up implementation study could assess barriers and facilitators to successful deployment, resources required, and cost-effectiveness; of note, many telehealth interventions have been shown to be more cost-effective than their in-person counterparts, which may increase scalability of TRAC [81, 82]. Future studies may also consider offering TRAC as an adjunct to medication for alcohol use disorder or other intensive addiction medicine treatment as a way of expanding the potential target population. Such studies could explore if and how the intervention increases feelings of self-efficacy and recovery capital [83] among individuals with alcohol use disorder.

## Conclusions

The TRAC intervention demonstrated feasibility, acceptability, and promising trends toward reducing alcohol use among VWH, though it was not powered to detect efficacy so those results should be interpreted with caution. The high retention and adherence rates, along with positive session ratings, highlight the intervention’s acceptability and potential for scalability as a remote, technology-based solution for problematic alcohol use among VWH. These findings provide a strong foundation for a future fully-powered randomized controlled trial to further evaluate the efficacy and broader applicability of the TRAC intervention.

## Data Availability

The datasets used and/or analyzed during the current study are available from the corresponding author on reasonable request.

## Abbreviations

VA: Department of Veteran’s Affairs
HIV: Human Immunodeficiency Virus
VWH: Veterans with HIV/AIDS
TRAC: Tracking and Reducing Alcohol Consumption
AUDIT: Alcohol Use Disorders Identification Test
